# Verification of the Reliability of an Automated Urine Test Strip Colorimetric Program Using Colorimetric Analysis: Survey Study

**DOI:** 10.2196/62772

**Published:** 2025-01-14

**Authors:** Keigo Inagaki, Daisuke Tsuriya, Takuya Hashimoto, Katsumasa Nakamura

**Affiliations:** 1 Clinical Nursing, Gerontological Nursing, Faculty of Nursing, Hamamatsu University School of Medicine, 1-20-1 Handayama, Chuo-ku, Hamamatsu city, Shizuoka, 431-3192, Japan, 81 053 435 2511; 2 Hamamatsu University School of Medicine, Hamamatsu City, Chuo-ku, Japan

**Keywords:** urine test strip, reliability, automatic urine analyzer, quasi-experimental study, colorimetric analysis, colorimetric, urinalysis, urinary, urine, evaluation, mobile phone

## Abstract

**Background:**

One method for noninvasive and simple urinary microalbumin testing is urine test strips. However, when visually assessing urine test strips, accurate assessment may be difficult due to environmental influences—such as lighting color and intensity—and the physical and psychological influences of the assessor. These complicate the formation of an objective assessment.

**Objectives:**

This study developed an “automated urine test strip colorimetric program” (hereinafter referred to as “this program”) to objectively assess urine test strips. Using this program may allow urine tests to be conducted at home. In this study, urine samples from hospitalized or outpatient patients were randomly obtained, and the reliability of this program was verified by comparing the agreement rate between this program and an automatic urine analyzer (US-3500 [Eiken Chemical Co, Ltd] and LABOSPECT 006 [Hitachi High-Tech Co, Ltd]). Furthermore, the sensitivity and specificity of the urine albumin test were investigated, and its applicability to screening for microalbuminuria was verified.

**Methods:**

A urine test strip was placed in a photography box with constant light intensity and color temperature conditions. The image taken with a smartphone camera on top of the photography box was judged by this program. This program used Accelerated KAZE to perform image-matching processing to reduce the effect of misalignment during photography. It also calculated and judged the item with the smallest color difference between the color chart and the urine test strip using the CIEDE2000 color difference formula. The agreement rate of the results of this program was investigated using the results of an automatic urine analyzer as the gold standard.

**Results:**

Compared with the judgments of an automatic urine analyzer, the average agreement rate for 12 items (protein, glucose, urobilinogen, bilirubin, ketone bodies, specific gravity, occult blood, pH, white blood cells, nitrite, creatinine, and albumin) was 78.6%. Furthermore, the average agreement rate of the 12 items within ±1 rank was 95.4%. The results showed a sensitivity of 100% and a specificity of 58.6% in determining albumin in urine, which is important for determining the stage of diabetic nephropathy. Finally, the area under the curve (0.907) derived from the receiver operating characteristic curve was satisfactory.

**Conclusions:**

The program developed by the authors can determine urine test strips without requiring calibration in a certain shooting environment. If this program can be used at home to perform urinary microalbumin tests, the early detection and treatment of diabetic nephropathy may prevent the condition from becoming severe.

## Introduction

Urinalysis testing displays vast physiological information and may be an effective tool for diagnosing various human diseases, including metabolic dysfunctions (eg, diabetes, urinary tract infections, and malignancies) and renal, cardiovascular, or hepatic disease [1-3]. Urinalysis test strips are an inexpensive, easy-to-use method that has long been used in clinical settings [4,5].

Urinalysis test strips change color in response to the concentration of the analyte present in the urine sample. Urinalysis test strips that react with urine analytes are checked against a reference color chart of several color blocks, each representing a specific analyte concentration.

The interpretation of colorimetric changes can be influenced by the health and psychological state of the observer [6,7]. Due to the lack of standardization and reading methods, it is not routinely used at home. The concept of self-screening at home has been progressively explored from multiple perspectives, and various types of devices have been developed depending on the application. For example, a Food and Drug Administration–approved product (Healthy.io) that uses a smartphone app to test items on urinalysis test strips for the early detection of chronic kidney disease and urinary tract infections is on the market. Various technologies exist for objectively judging urine test strips using smartphone apps such as “Vivoo” and “Uchek.”

This study developed an automated urine test strip colorimetric program to automatically judge urinalysis test strips from smartphone images [8]. This program automatically identified the item with the most negligible color difference between the reference color chart and urinalysis test strip and displayed the urinalysis results. Since the assessment was unaffected by the measurer’s physical condition or psychological state, the proposed method provided an objective rather than a subjective visual evaluation.

This study verified the reliability of an automated urine test strip colorimetric program, investigated the sensitivity and specificity of urinary albumin testing, and verified its applicability for microalbuminuria screening. Using real patient urine specimens, the concordance rate between creatinine and albumin and the 10 previously validated items was assessed. The concordance rate for each judgment item and the sensitivity and specificity of albumin were further investigated.

## Methods

### Overview

**Figure 1.**
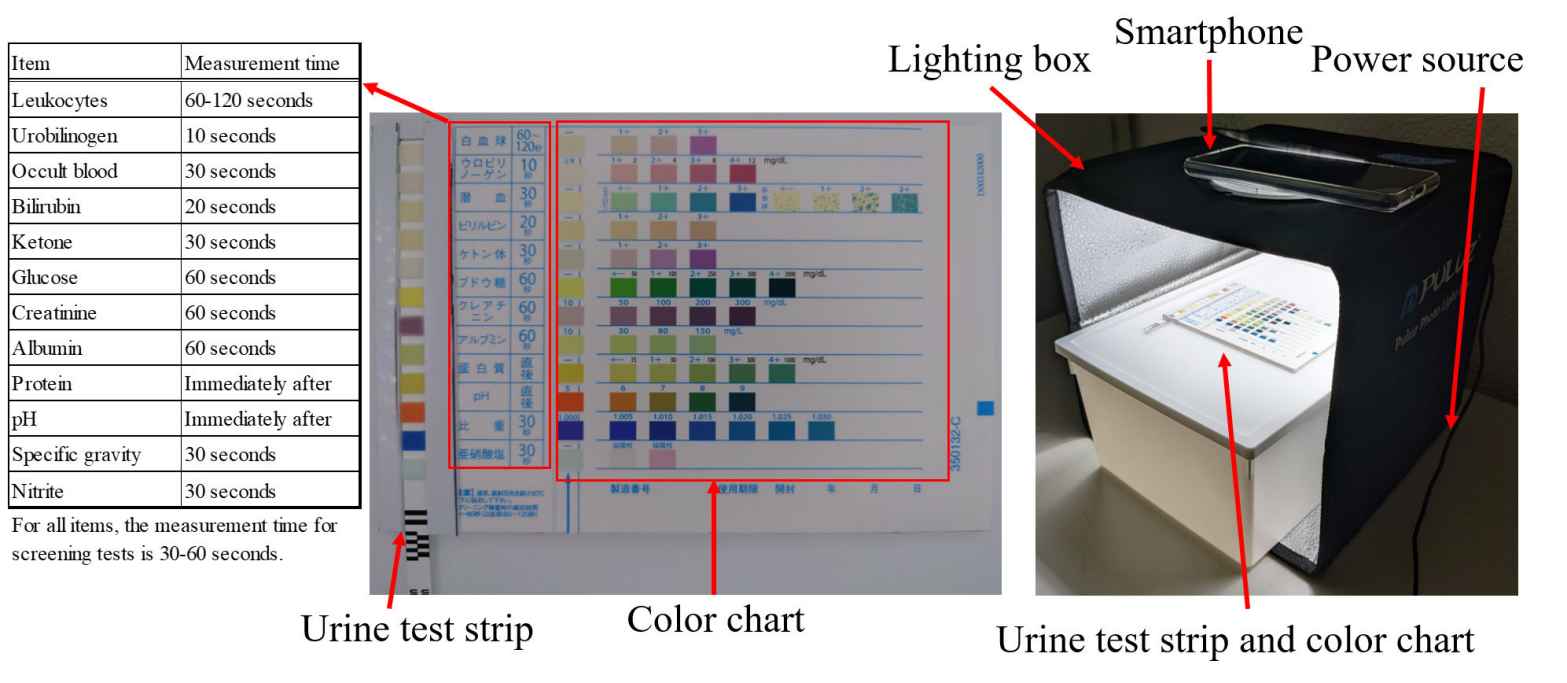
Reference color chart and shooting environment used for color comparison of urine test strips.

Figure 1 depicts this study’s experimental environment. A photography box (PU5025B; PULUZ) was used to maintain a constant lighting environment. An Xperia 5II (XQ-AS42, SONY) smartphone was used. The smartphone had a standard triple-lens camera (ZEISS lens) with a 1/1.7-inch large format sensor, approximately 12.2 million pixels/24-mm focal length, and a 1.7/dual photodiode/hybrid image stabilization (optical+ electronic) *F* value. A urinalysis test strip (Uropaper III Eiken [E-UR97]; Eiken Chemical Co, Ltd) capable of testing 12 items was used as the test strip. The reference color chart, commonly used in clinical practice, was used as a comparison target for the urinalysis test strips (Figure 1). From June 2022 to January 2023, 1184 urine specimens were randomly extracted from specimens handled by the laboratory department of a hospital in Hamamatsu City. The inclusion criteria were urine samples from people aged 20-90 years who were evaluated at the laboratory department of a hospital in Hamamatsu City. The exclusion criteria were urine samples from people admitted to the pediatric ward and people with cloudy urine or gross hematuria. Basic information on the specimens is shown in Table 1. The US-3500 (Eiken Chemical Co, Ltd) and LABOSPECT 006 (Hitachi High-Tech Co, Ltd) were used as automatic urine analyzers for comparison with the results of the proposed method. The US-3500 was used to compare urobilinogen, occult blood, protein, glucose, ketone bodies, bilirubin, nitrite, specific gravity, white blood cells, and pH, and the LABOSPECT 006 was used to compare creatine and albumin levels. All 1184 specimens of urobilinogen, occult blood, protein, glucose, ketone bodies, bilirubin, nitrite, specific gravity, and pH were obtained using an automatic analyzer. Automated analyzer data were obtained for 1182 leukocyte, 214 creatine, and 42 albumin samples. The automatic urine test strip colorimetric program developed in this study was created using a desktop computer (HP EliteDesk 800 G4 TWR, HP), with Python as the development language and OpenCV library for image processing.

**Table 1.** Basic information on 1184 urine samples randomly extracted from specimens handled by the testing department of a hospital in Hamamatsu City from June 2022 to January 2023.

Content	Individuals (N=1184)
**Sex, n (%)**	
Female	648 (54.7)
Male	536 (45.3)
**Sex, Average age (SD)**	
Female	48.16 (21.4)
Male	58.00 (22.8)
**Clinical department, n (%)**	
Department of rehabilitation	1 (0.1)
General surgery	12 (1)
Lower gastrointestinal surgery	14 (1.2)
Liver internal medicine	36 (3)
Hepato-biliary-pancreatic surgery	3 (0.3)
Ophthalmology	57 (4.8)
Hematology	55 (4.6)
Vascular surgery	1 (0.1)
Respiratory surgery	8 (0.7)
Respiratory medicine	31 (2.6)
Obstetrics and gynecology	263 (22.2)
Oral surgery	5 (0.4)
Otorhinolaryngology	6 (0.5)
Cardiology	24 (2)
Pediatrics	86 (7.3)
Gastroenterology	23 (1.9)
Upper gastrointestinal surgery	53 (4.5)
Cardiovascular surgery	18 (1.5)
Nephrology	73 (6.2)
Orthopedics	66 (5.6)
Psychiatry	4 (0.3)
Endocrinology and metabolism	131 (11.1)
Breast surgery	5 (0.4)
Neurosurgery	10 (0.8)
Urology	169 (14.3)
Dermatology	14 (1.2)
Immunology/rheumatology	15 (1.3)
Clinical pharmacology	1 (0.1)

### Experimental Methods

#### Judgment of the Automatic Urine Dipstick Colorimetric Program

##### Automated Urine Dipstick Colorimetric Program

**Figure 2.**
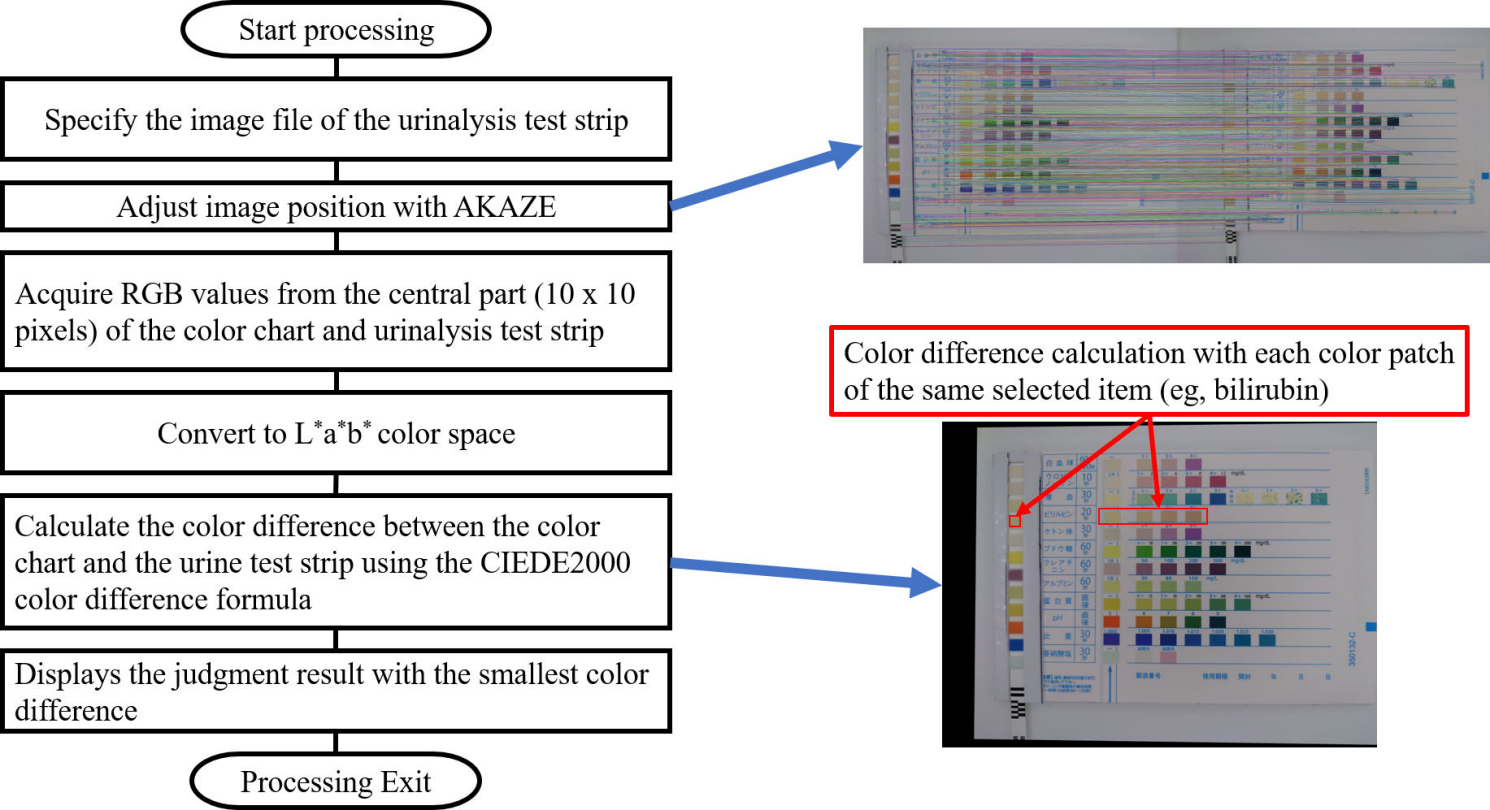
Process flow for comparing the color of urine test strips and color charts. RGB: red, green, and blue.

Figure 2 shows the processing flow of the automatic urine dipstick colorimetric program. The colors of the test paper for the urine test and color chart were compared according to the following procedure:

1. Specify the image file of the urinalysis strip.
2. Adjust the image position using Accelerated KAZE (AKAZE).
3. Acquire RGB (red, green, and blue; from the RGB color model) values from the central part (10×10 pixels) of the color chart and urinalysis test strip.
4. Convert to the L*a*b* color space.
5. Calculate the color difference between the color chart and the urine test strip using the CIEDE2000 color difference formula (color difference calculation with each color patch of the same selected item).
6. Display the judgment results with the smallest color difference.

##### Filming Environment and Procedures

The color was evaluated using the following procedure in the same environment using a photography box (PU5025B):

1. Turn on the shooting box. Leave for 30 minutes to stabilize the lighting.
2. Start shooting 30 minutes after turning on the power.
3. Install the smartphone with the camera activated on top of the shooting box. Set the camera color temperature to sunlight, which is the same as that of the lighting of the shooting box.
4. Soak the urine test strip completely in the urine (approximately 1 or 2 seconds).
5. Lightly touch the side of the test paper with the tissue paper to remove excess urine on the test paper.
6. Place the test paper in a shooting box.
7. Take a picture 50 seconds after removing it from the urine sample (ie, when the paper is removed from the Spitz).
8. Judge the photographed images using an automatic urine dipstick colorimetric program.
9. Acquire age, sex, clinical department, and urinalysis results from electronic medical records.
10. Match the results with those of the automatic urine analyzer.

### Accelerated KAZE

In this study, AKAZE, which is available in the OpenCV library, was used in image matching to reduce the effects of misalignment during shooting. AKAZE is an accelerated version of the algorithm used in KAZE [9], which is a 2D feature detection and description algorithm in nonlinear scale space developed by Alcantarilla et al [10]. This feature extraction algorithm is robust to changes in scaling, rotation, and lighting, and is similar to scale-invariant feature transform (SIFT). The KAZE algorithm is an improved version of SIFT and uses a nonlinear diffusion filter instead of a Gaussian filter to extract features while preserving the original image features and edges. SIFT uses a Gaussian filter to detect features. The center pixel of interest to be processed has the largest weight, and the outer pixels have smaller weights. This allows for images to be blurred while retaining more information around the pixels.

### Judging Method for Urine Test Strips

The following methods are used for judging:

1. Approximate selection method: select the color on the color chart that is closest to the color of the test strip.
2. Rounding down method: if the color of the test strip does not reach the color on the color chart, judge it as a color with a lower density.
3. Rounding up method: if the color of the test strip is even slightly darker than that of the color chart, judge it as a color with a higher density.

Currently, there is no standard judgment method, but the Urinary Protein Measurement Committee has reported that “The approximate selection method seems to be theoretically correct, but it is prone to differences due to lighting and differences in each person’s vision” [11]. Therefore, if the effects of lighting and individual differences can be reduced using this program, the “approximate selection method” may be suitable as an evaluation method. In this study, the “approximate selection method” was used.

### Statistical Analysis

The rate of agreement was calculated using the results of the automatic urine analyzer. A concordance rate of ±1 was calculated as the concordance rate within 1 rank before and after the matching item. IBM SPSS Statistics 28th edition was used for the receiver operating characteristic (ROC) curve and area under the curve (AUC).

### Ethical Considerations

This research was reviewed and approved by the ethics committee of the Hamamatsu University School of Medicine (approval number: 21‐239). This study used existing samples in addition to existing information. However, as the samples were submitted to outpatient and other clinics, it was difficult to obtain direct consent. As such, information about this study, including the purpose of use of the samples and information, was made public on the Hamamatsu University School of Medicine’s website and posted on notices within the hospital. Research subjects were given an opt-out form via information disclosure document and were guaranteed the opportunity to refuse participation in the study.

The samples and information provided, including personal information, were managed responsibly by the principal investigator (KI) and were anonymized such that specific individuals could be identified using a correspondence table or other means. This study collected only existing samples and information accumulated in the course of normal medical treatment and care, and there were no adverse events or malfunctions caused by the implementation of the study. This study targeted only the collection of existing samples and information accumulated during routine medical treatment and care, and there was no possibility of health damage occurring as a result of the study, so compensation for health damage and insurance enrollment did not apply.

## Results

### Agreement Rates and ±1 Rank Agreement Between Automated Urine Analyzers and Automated Urine Test Paper Colorimetric Programs

In this study, the US-3500, which is capable of qualitative evaluation using urine test strips, and the LABOSPECT 006, which is capable of quantitative evaluation of creatinine and albumin, were used. The results of the automated urine analyzers (US-3500 and LABOSPECT 006) were referred to the values recorded in the electronic medical records of the cooperating hospitals in the study. Table 2 shows the concordance rate of the automated urine analyzer and automated urine test strip colorimetric program, a concordance rate of ±1 rank, and the number of specimens. Urobilinogen, occult blood, protein, glucose, ketone bodies, bilirubin, nitrite, specific gravity, and pH were evaluated for concordance in 1184 samples. For white blood cells, 1182 specimens were evaluated for concordance. The amount of data obtained for creatinine and albumin is small due to the small number of daily test requests at the hospitals cooperating in the study. Therefore, the concordance rate was evaluated using 214 specimens for creatine and 42 specimens for albumin, which was in line with the data obtained from the automatic urine analyzer. The concordance rate for each item was more than 60%, except for specific gravity. The mean concordance for the 12 items was 78.6%. The matching rate of the ±1 rank for each item exceeded 90% for items other than specific gravity. The mean ±1 rank agreement for the 12 items was 95.4%. Multimedia Appendix 1 shows the concordance rates for each inspection item. The results indicate that an automated urine test paper colorimetric program could be applied to screening within ±1 rank.

**Table 2.** The concordance and ±1 rank match rate of the output results of the automated urine analyzer and the automated urine test strip colorimetric program and the number of samples obtained^a^.

Item	Concordance rate, %	±1 rank match rate, %	Number of samples, n
Protein	83.1	100	1184
Glucose	94.8	100	1184
Urobilinogen	86	99.9	1184
Bilirubin	72.4	99.4	1184
Ketone	98	100	1184
Specific gravity	14.4	49.7	1184
Occult blood	87.8	97.8	1184
pH	80.7	100	1184
Leukocytes	86.6	99.1	1182
Nitrite	98.5	100	1184
Creatinine	64	98.6	214
Albumin	76.2	100	42
Average of 12 items	78.6	95.4	N/A^b^

a The samples were randomly selected from samples handled in the testing department of a hospital in Hamamatsu City from June 2022 to January 2023.

b N/A: not applicable.

### Sensitivity and Specificity for Albumin

**Figure 3.**
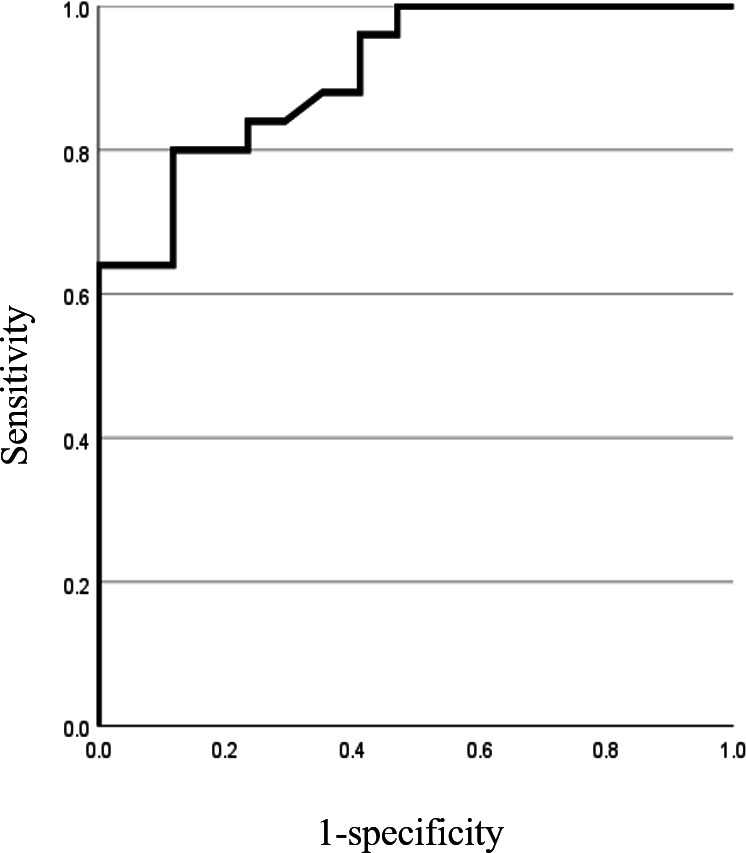
Receiver operating characteristic curve showing sensitivity and specificity of albumin. Patients with albumin levels of ≥30 were classified as those with diabetes, and those with albumin levels <30 were classified as patients with no diabetes. Sensitivity and specificity were calculated by creating a cross-table. From June 2022 to January 2023, 42 urine specimens were extracted from specimens handled by the laboratory department of a hospital in Hamamatsu City.

Trace albumin is a useful biomarker for early detection of nephropathy in patients with diabetes. Therefore, if trace albumin, which is important in determining the second stage of nephropathy, can be detected, it may lead to early detection of the progression of nephropathy. To confirm the applicability of our automated urine test paper colorimetric program for screening trace albuminuria, we calculated the sensitivity and specificity of the program for albumin. Since this program is a qualitative evaluation using urine test strips, the albumin value cannot be corrected for creatinine. Therefore, only the albumin value in urine was used for validation. Table 3 shows a cross-table for calculating the sensitivity and specificity of albumin. A diagnostic factor for diabetic nephropathy is microalbuminuria, which ranges from 30 to 299 mg/L. The cross-table was created assuming that patients with albumin levels of ≥30 and <30 were classified as having and not having the disease. Program judgment results show the program’s output; microalbumin (urine automatic analysis data) shows the results recorded by LABOSPECT 006 in the electronic medical records of the collaborating hospitals. Cross-tab calculations yielded a sensitivity and specificity of 100% and 58.6%, respectively. Figure 3 shows the ROC curves. The AUC was 0.907, which is very favorable. Therefore, the results indicate that this program could be applied to screening for stage 2 nephropathy in diabetic nephropathy.

**Table 3.** Albumin sensitivity and specificity^a^.

	Microalbumin (urine automatic analysis data)		Total
	With disease (albumin ≥30 mg/L)	With no disease (albumin <30 mg/L)	
**Program judgment result**			
Positive (albumin ≥30 mg/L)	13	12	25
Negative (albumin <30 mg/L)	0	17	17
Total	13	29	

a Patients with albumin levels of ≥30 and <30 were classified as those with diabetes and with no diabetes, respectively. Sensitivity and specificity were calculated by creating a cross-tabulation using the output of the automated urine analyzer and the automated urine test strip colorimetric program. From June 2022 to January 2023, 42 urine specimens were extracted from specimens handled by the laboratory department of a hospital in Hamamatsu City.

## Discussion

### Principal Results

This study used real patient urine specimens to investigate the agreement between an automated urine test strip colorimetric program and an automated urine analyzer. The concordance rate for each judgment item and the sensitivity and specificity of albumin were also evaluated. The concordance rate of the ±1 rank was 90% or more; thus, the concordance rate could be improved by devising the judgment threshold and judgment algorithm of the program.

The concordance rate of specific gravity obtained with this program was very low and was consistent with previous studies [12]. In the visual judgment, the colors of the reference color chart and test paper for urinalysis differed. Determining specific gravity with a low matching rate by simple color comparison was challenging; therefore, a determination algorithm should be considered.

The detailed results for each item showed many negative and few positive judgments. However, despite the small number of positive tests, many items matched, supporting the effectiveness of the proposed method.

The proposed method determined the matching rate using a program that identifies the color closest to the reference color chart. Since the proposed method used the “approximate selection method,” some items may present a low matching rate. Therefore, other evaluation methods should be considered in the future.

Previous reports have included many studies evaluating the reliability of programs using real urine samples, artificial urine, and reagents [13-17]. In a report using real urine samples, urine testing was performed using a smartphone app developed by Healthy.io, and its acceptability and feasibility were evaluated [16,17]. The satisfaction rate was very high among patients and caregivers, and the app could greatly enhance patient-centered care [16]. The potential of remote digital urine testing has also been reported [17].

Rahmat et al [18] validated the scanner color comparison using real urine specimens and reported an accuracy of 95.45% using a high-resolution scanner. The low-resolution scanner reported 83%, comparable with our automated urine test strip colorimetric program. Africa and Velasco [19] also verified the accuracy of color comparison using 45 samples from an accredited urinalysis laboratory and reported an accuracy of 96.519%. However, besides evaluating 10 items (ie, protein, glucose, ketone, specific gravity, occult blood, leukocytes, nitrite, urobilinogen, bilirubin, and pH), these 2 studies did not provide the breakdown of the concordance rate (ie, negative and positive breakdown and item-by-item classification). Urinary albumin and creatinine levels, necessary for determining diabetic nephropathy, have not yet been evaluated in the literature. Determining albumin and creatinine may help screen for microalbuminuria, enabling intervention before diabetic nephropathy becomes severe.

The sensitivity and specificity of this method for albumin detection were 100% and 58.6%, respectively. These suggest potentially high and low rates of false positives and negatives, respectively, allowing for people with diseases to be identified. Furthermore, the AUC derived from the ROC curve was 0.907, indicating satisfactory results. Therefore, the proposed method may be used as initial screening.

The proposed method may also support point-of-care (POC) testing at home to prevent the progression of diabetic nephropathy. Thakur et al [15] reported a method for albumin screening using smartphones and urine test strips. The study reported a 92% accuracy rate, validated only with reagents. In contrast, this study used actual urine specimens, and excellent screening results were obtained with a concordance rate of 76.2%, sensitivity of 100%, and specificity of 58.6%, relative to the results of an automated urine analyzer. Due to the COVID-19 pandemic, many people refrained from seeing doctors. As such, the proposed method is expected to be particularly useful for POC tests for home health management. Therefore, we are currently developing an application that uses the proposed system.

The proposed automatic urine test strip colorimetric program focuses only on color differences but does not require a large amount of data from machine learning. In addition, if standard color chart data from each manufacturer become available, urinalysis test strips from various manufacturers worldwide may be used without requiring calibration. In the future, the accuracy of determination may be improved by using data obtained from specimens, adjusting the threshold for determination, and examining determination methods.

### Limitations

Despite its contributions, this study has limitations. The results do not apply to all smartphones because verification was performed using only 1 smartphone model. In the future, the program should be tested on other operating systems, smartphones, and cameras. In addition, owing to lighting limitations due to the shooting box, development did not progress sufficiently enough that judgment could be formulated with just a smartphone. This system is not suitable as a POC test; therefore, future investigations should verify whether similar results can be obtained using the flash function of a smartphone. In the future, the differences between multiple models and methods that do not use shooting boxes should be considered.

In addition, this study used urine test strips to evaluate the agreement between the white blood cell count obtained by an automated urine analyzer and the program developed in this study. Normally, the agreement with sediment analysis is verified. Future studies should compare the results of the formulated program with those from sediment analysis to compare the accuracy.

When urine samples were randomly collected, many samples did not have albumin analysis in the doctor’s orders. Therefore, the albumin results from the automated urine analyzer were not recorded in the electronic medical record, and the match rate could not be calculated for many samples. The study protocol should be adjusted so that an adequate sample of albumin tests can be collected.

Normally, creatinine should be corrected in urinalysis results. However, given that the coincidence rate of creatinine was not high, only albumin was highlighted. In the future, accuracy can be improved by evaluating the albumin-creatinine ratio.

The analyzers used in this study (US-3500 and LABOSPECT 006) can evaluate reflectance, allowing for a more accurate evaluation of the consistency rate of urine test strips. However, this study used data stored in electronic medical records, and reflectance data could not be obtained. In the future, reflectance data should also be analyzed.

In this study, the agreement rate by qualitative evaluation was verified using urine test strips. However, sensitivity is low when using urine test strips. Therefore, in the future, reliability should be verified by comparing data that can be quantitatively measured, such as immunoturbidimetric methods. Finally, to avoid over- or underestimating clinical scenarios, sample collection periods should span several years.

### Conclusions

This study used real patient urine specimens to investigate the agreement between an automated urine test strip colorimetric program and an automated urine analyzer. It also investigated the concordance rate for each judgment item and the sensitivity and specificity of the program in evaluating albumin. The average concordance rate and rate of ±1 rank for the mean of the 12 inspected items were 78.6% and 95.4%, respectively. These results clarified that colorimetric analysis in a fixed imaging environment could be used to determine urine test strips without calibration.

Urinary albumin determination, essential for determining the stage of diabetic nephropathy, resulted in a sensitivity of 100% and a specificity of 58.6%. In addition, the AUC derived from the ROC curve was 0.907, indicating good results. The proposed method may be used for the screening of microalbuminuria to help prevent the exacerbation of diabetic nephropathy.

## Supplementary material

10.2196/62772 Multimedia Appendix 1 Supplementary Table

## Abbreviations

AKAZE: Accelerated KAZE
AUC: area under the curve
POC: point-of-care
RGB: red, green, and blue
ROC: receiver operating characteristic
SIFT: scale-invariant feature transform
