# Supplementary material for: Verification of the Reliability of an Automated Urine Test Strip Colorimetric Program Using Colorimetric Analysis: Survey Study
Source: JMIR Form Res. 2025 Jan 14;9:e62772. doi: 10.2196/62772 (PMC11750113; doi:10.2196/62772)
Supplement: Multimedia Appendix 1 [file formative-v9-e62772-s001.docx]

**Supplementary Table. Concordance rates for each inspection item**

**The vertical and horizontal axes represent the program and cross-tabulation table of the determination results of the automatic urine analyzer, respectively. Colored squares in the cross-tabulation table are items for which the program results and automatic urine analyzer matched.**

Protein results (N=1184)

| Program judgment result | 4+ | 0 | 0 | 0 | 0 | 2 | 2 |
| --- | --- | --- | --- | --- | --- | --- | --- |
|  | 3+ | 0 | 0 | 0 | 4 | 8 | 0 |
|  | 2+ | 0 | 0 | 9 | 18 | 0 | 0 |
|  | 1+ | 0 | 24 | 84 | 3 | 0 | 0 |
|  | ± | 145 | 127 | 9 | 0 | 0 | 0 |
|  | - | 745 | 4 | 0 | 0 | 0 | 0 |
|  |  | - | ± | 1+ | 2+ | 3+ | 4+ |
|  |  | Judgment result of automatic urine analyzer | | | | | |

Glucose results (N=1184)

| Program judgment result | 4+ | 0 | 0 | 0 | 0 | 35 | 61 |
| --- | --- | --- | --- | --- | --- | --- | --- |
|  | 3+ | 0 | 0 | 0 | 4 | 4 | 1 |
|  | 2+ | 0 | 0 | 10 | 10 | 1 | 0 |
|  | 1+ | 0 | 3 | 13 | 0 | 0 | 0 |
|  | ± | 0 | 20 | 3 | 0 | 0 | 0 |
|  | - | 1015 | 4 | 0 | 0 | 0 | 0 |
|  |  | - | ± | 1+ | 2+ | 3+ | 4+ |
|  |  | Judgment result of automatic urine analyzer | | | | | |

Urobilinogen results (N=1184)

| Program judgment result | 4+ | 0 | 0 | 1 | 0 | 0 |
| --- | --- | --- | --- | --- | --- | --- |
|  | 3+ | 0 | 0 | 4 | 0 | 0 |
|  | 2+ | 0 | 21 | 1 | 0 | 0 |
|  | 1+ | 139 | 29 | 1 | 0 | 0 |
|  | normal(±) | 988 | 0 | 0 | 0 | 0 |
|  |  | normal(±) | 1+ | 2+ | 3+ | 4+ |
|  |  | Judgment result of automatic urine analyzer | | | | |

Bilirubin results (N=1184)

| Program judgment result | 3+ | 0 | 0 | 1 | 1 |
| --- | --- | --- | --- | --- | --- |
|  | 2+ | 7 | 3 | 1 | 0 |
|  | 1+ | 316 | 1 | 0 | 0 |
|  | - | 854 | 0 | 0 | 0 |
|  |  | - | 1+ | 2+ | 3+ |
|  |  | Judgment result of automatic urine analyzer | | | |

Ketone results (N=1184)

| Program judgment result | 3+ | 0 | 0 | 2 | 4 |
| --- | --- | --- | --- | --- | --- |
|  | 2+ | 0 | 5 | 11 | 0 |
|  | 1+ | 16 | 21 | 1 | 0 |
|  | - | 1124 | 0 | 0 | 0 |
|  |  | - | 1+ | 2+ | 3+ |
|  |  | Judgment result of automatic urine analyzer | | | |

Leukocytes results (N=1182)

| Program judgment result | 3+ | 0 | 0 | 13 | 101 |
| --- | --- | --- | --- | --- | --- |
|  | 2+ | 1 | 16 | 71 | 17 |
|  | 1+ | 48 | 44 | 18 | 2 |
|  | - | 808 | 35 | 7 | 1 |
|  |  | - | 1+ | 2+ | 3+ |
|  |  | Judgment result of automatic urine analyzer | | | |

Nitrite results (N=1184)

| Program judgment result | Positive | 13 | 32 |  |  |
| --- | --- | --- | --- | --- | --- |
|  | Negative | 1134 | 5 |  |  |
|  |  | Negative | Positive |  |  |
|  |  | Judgment result of automatic urine analyzer | | | |

Occult Blood results (N=1184)

| Program judgment result | 3+ | 0 | 0 | 0 | 10 | 61 |
| --- | --- | --- | --- | --- | --- | --- |
|  | 2+ | 0 | 0 | 3 | 23 | 9 |
|  | 1+ | 0 | 1 | 28 | 17 | 5 |
|  | ± | 16 | 51 | 21 | 9 | 2 |
|  | - | 877 | 41 | 8 | 1 | 1 |
|  |  | - | ± | 1+ | 2+ | 3+ |
|  |  | Judgment result of automatic urine analyzer | | | | |

pH results (N=1184)

| Program judgment result | 9 | 0 | 0 | 0 | 1 | 0 |
| --- | --- | --- | --- | --- | --- | --- |
|  | 8 | 0 | 0 | 88 | 31 | 0 |
|  | 7 | 0 | 47 | 270 | 1 | 0 |
|  | 6 | 68 | 390 | 12 | 0 | 0 |
|  | 5 | 265 | 11 | 0 | 0 | 0 |
|  |  | 5 | 6 | 7 | 8 | 9 |
|  |  | Judgment result of automatic urine analyzer | | | | |

Specific Gravity results (N=1184)

| Program judgment result | 1.030 | 1 | 0 | 1 | 40 | 99 | 101 | 74 |
| --- | --- | --- | --- | --- | --- | --- | --- | --- |
|  | 1.025 | 0 | 0 | 7 | 82 | 50 | 16 | 15 |
|  | 1.020 | 0 | 21 | 139 | 107 | 31 | 9 | 13 |
|  | 1.015 | 8 | 173 | 109 | 23 | 7 | 7 | 3 |
|  | 1.010 | 0 | 0 | 0 | 0 | 0 | 0 | 0 |
|  | 1.005 | 18 | 26 | 2 | 0 | 0 | 0 | 1 |
|  | 1.000 | 1 | 0 | 0 | 0 | 0 | 0 | 0 |
|  |  | 1.000 | 1.005 | 1.010 | 1.015 | 1.020 | 1.025 | 1.030 |
|  |  | Judgment result of automatic urine analyzer | | | | | | |

Creatinine results (N=214)

| Program judgment result | 300 | 0 | 0 | 0 | 2 | 2 |
| --- | --- | --- | --- | --- | --- | --- |
|  | 200 | 0 | 0 | 2 | 4 | 3 |
|  | 100 | 0 | 2 | 24 | 16 | 0 |
|  | 50 | 5 | 82 | 29 | 1 | 0 |
|  | 10 | 25 | 15 | 2 | 0 | 0 |
|  |  | 10 | 50 | 100 | 200 | 300 |
|  |  | Judgment result of automatic urine analyzer | | | | |

Albumin results (N=42)

| Program judgment result | 150 | 0 | 0 | 0 | 3 |
| --- | --- | --- | --- | --- | --- |
|  | 80 | 0 | 0 | 2 | 0 |
|  | 30 | 9 | 10 | 1 | 0 |
|  | 10 | 17 | 0 | 0 | 0 |
|  |  | 10 | 30 | 80 | 150 |
|  |  | Judgment result of automatic urine analyzer | | | |
